# Waste to Wealth Strategy: Preparation and Properties of Lightweight Al_2_O_3_-SiO_2_-Rich Castables Using Aluminum Dross Waste

**DOI:** 10.3390/ma14247803

**Published:** 2021-12-16

**Authors:** Nan Su, Zishen Li, Youdong Ding, Hongliang Yang, Jingzhou Zhang, Gaofeng Fu

**Affiliations:** 1 Key Laboratory for Ecological Metallurgy of Multimetallic Mineral, Ministry of Education, Northeastern University, Shenyang 110819, China; 18701145786@163.com (N.S.); Lishen990213@foxmail.com (Z.L.); dingyoudong_2006@126.com (Y.D.); yangneu349@163.com (H.Y.); zjz19960905@163.com (J.Z.); 2 School of Metallurgy, Northeastern University, Shenyang 110819, China; 3 Sinosteel Equipment & Engineering Co., Ltd., 8 Haidian St., Beijing 100080, China

**Keywords:** aluminum dross waste, castable, thermal conductivity, mechanical properties

## Abstract

Aluminum dross is a well-known industrial waste generated in the aluminium industry, and its recycling and reuse is still a worldwide issue. Herein, aluminum dross waste (ADW) was recycled to progressively replace the aggregate fraction of clay at 70, 75, 80, 85, and 90 wt% for the fabrication of Al_2_O_3_-SiO_2_-rich porous castable refractories. Their physical properties and mechanical behavior were assessed by the measurement of linear shrinkage rate, bulk density, apparent porosity, cold crushing strength, and thermal conductivity. The microstructure and phase evolutions were analyzed via scanning electron microscopy (SEM) and X-ray diffraction (XRD). The incorporation of 85 wt% of ADW allowed the development of a waste-containing conventional refractory castable with improved properties as compared to those of the other samples. The sustainable refractory castable exhibited decent thermal conductivity and physical and mechanical characteristics, and is suitable for application as reheating furnace lining. It is a “green” practice to partially replace the traditional raw materials with industrial waste in the manufacture of conventional refractory castables and provides environmental and economic benefits.

## 1. Introduction

Owing to their unique advantages in workability, cost efficiency, energy-saving, and structural integrity, refractory castables have found increasing applications in the pyrometallurgical, cement, and chemical industries [1]. Among them, the Al_2_O_3_-SiO_2_ refractory castable is a popular choice that has been widely employed in the steelmaking industry for linings and covers [2]. It is well known that the aggregate is a major component of the ceramic body, and its choice considerably influences the properties of refractory castables [3]. In general, due to the advantages of low-cost, easy availability, and abundance, natural sources, such as clays, kaolinites, sillimanites, and bauxites, are the most common aggregates used for Al_2_O_3_-SiO_2_ refractory castables [4]. However, both the growth in industrial production and the increase in natural consumption have led to a rapid decline in the availability of natural sources. For instance, about 340 billion tons of clay are being excavated per year worldwide [5,6]. The ceramic industry faces weakening and depleting mineral and non-mineral deposits, which increases ecological problems and costs of mineral raw materials [7]. Therefore, it is a “green” practice to partially replace traditional raw materials with industrial wastes in the manufacturing of conventional refractory, which provides environmental and economic benefits [8].

On the other hand, with the exponential growth of aluminum metal and aluminum-related products, the production of aluminum dross waste (ADW) has also increased. ADW is formed on the surface of molten aluminum that is exposed to the furnace atmosphere during primary and secondary aluminum fusion processing [9]. For 100 kg of molten aluminum, 15–25 kg of ADW, on average, is generated [10]. Approximately 5 million tons of ADW is generated annually throughout the world and the majority of this waste is disposed via land filling [11]. This method of disposal leads to issues, such as loss of valuable metals, emission of harmful gases when it comes in contact with water, and leaching of toxic metal ions into ground water [12]. Disposal and recycling of ADW produced during aluminum melting is a worldwide problem. Recycling presents a strategy to minimize waste with benefits such as reducing the demand for new resources, transportation costs, and production energy costs [13]. The ADW is usually a mixture of free Al metal, nonmetallic substances, such as aluminum oxide, nitride, and carbide, salts, and metal oxides [14]. Currently, most of the research works have focused on recycling Al from ADW through physical and chemical methods, for instance, heating the dross in a rotating furnace using a molten salt flux for the separation of molten Al from solid oxide fraction and protection of Al metal against oxidation [15]. However, large amounts of water-leachable salts and other elements and compounds that may have deleterious effects on the environment are produced in the process.

Meanwhile, some ceramics, refractories, and glasses were successfully prepared based on the chemical compositions of ADW. López et al. [16] synthesized calcium alu-minates from the non-saline dross produced during manufacturing metallic aluminum in holding furnaces. Romero et al. [17] sintered glass-ceramic tiles by the sinter-crystallization of mixtures composed of aluminum slag and reclaimed packaging glass. Shen et al. [18] manufactured soda-lime-aluminosilicate (SLAS) glass-ceramics with high alumina contents (over 30 wt%) using secondary aluminum dross without additional nucleation agents. Ewais et al. [19] used ADW and rutile ore powders as preliminary materials and successfully prepared magnesium aluminum titanate (MAT) based ceram-ics by reaction sintering at a temperature of 1300 °C. El-Amir et al. [20] introduced a new method for producing sustainable foam glasses from soda-lime glass powders using ADW as a foaming agent.

This research aims to use ADW generated from aluminum industries as a partial replacement for high-in-demand traditional raw material of clay that is used in lightweight Al_2_O_3_-SiO_2_ refractory castables with promising applications in the steelmaking industry. The variations of physical and mechanical properties, such as bulk density, apparent porosity, cold crushing strength, and thermal conductivity have been investigated via phase and microstructure analysis.

## 2. Materials and Methods

ADW and clay were used as raw materials for the preparation of aggregates with sizes of 6–3 mm, 3–1 mm, and 1–0 mm, respectively. The chemical compositions of ADW and clay were tested by X-ray fluorescence (XRF, ZSX Primus II; Rigaku, Tokyo, Japan) and are listed in Table 1. Table 2 presents the composition of designed experimental refractory samples. The ADW% that replaced clay in aggregates in all the cases is represented by 70A, 75A, 80A, 85A, and 90A. Aggregates with ADW powders, clay, analytically pure alumina powder (CL370; Al_2_O_3_ ≥ 95%, Hengjia, Guangzhou, China) and calcium aluminate cement (CAC, CA70, Zhengzhou, China) were used as starting materials and binder, respectively. The raw materials (see Table 1) were successively dry- and wet-mixed in a laboratory mixer for 10 min. The mixed castables were immediately cast into molds of 25 mm × 25 mm × 25 mm and 25 mm × 25 mm × 150 mm with vibration. The specimens were cured at room temperatures for 24 h. After demolding, the samples were dried at 110 °C for 24 h and then sintered at 900–1300 °C with a 5 K/min heating rate. After heating at the designed temperature for 2 h, the samples were naturally cooled to room temperature in the furnace.

Bulk density (Equation (1)) and apparent porosity (Equation (2)) of the castable were measured using the Archimedes principle with the deionized water as the medium (homemade equipment) [21]. The cold crushing strengths (CCS, Equation (3)) of the samples were measured using a compression testing machine at a constant rate of load of 0.5 mm/min (Constant Hydraulic Machinery Co., Ltd., Zaozhuang, China). Linear change rates of samples were calculated by measuring lengths of samples before and after sintering (Equation (4)). Thermal conductivities of samples were measured via the water flow plate method (YB/T4130-2005, PBD-02P, PRECONDAR, Luoyang, China) and calculated using Equation (5). Phase analysis of the samples was conducted using an X-ray diffractometer (XRD, D8 Advance, Bruker AXS, Berlin, Germany) operating with Cu Kα (λ = 0.154056 nm) radiation source. The scan range was 2*θ* = 10–90° at the scan rate of 8° per minute (scan step: 0.02°, exposure time for step: 0.15 s, voltage: 40 KV, and current: 40 mA). The microstructural development of the samples was studied using a scanning electron microscope (SEM, QuantaFEG250, FEI, Hillsboro, OR, USA) with the support of energy-dispersive X-ray (EDX), and the accelerating voltage was 20 kV.

(1) $$D=\frac{m_{0}D_{l}}{m_{2}-m_{1}},$$

(2) $$q=\frac{m_{2}-m_{0}}{m_{2}-m_{1}},$$

where *D* and *q* are the bulk density (g∙cm^−3^) and apparent porosity (%) of the refractories. *m*_0_ is the mass of refractories in air (g), *m*_1_ is the mass of refractories in the water (g), *m*_2_ is mass of refractories with free bubbles on the surface (g), and *D*_l_ is the density of water (1.0 g∙cm^−3^).

(3) $$C=\frac{F}{A},$$

where *C* is the cold crushing strength, *F* is the force imposed on the samples, and *A* is the sample’s area.

(4) $$L_{f}=\frac{L_{2}-L_{1}}{L_{1}}\times 100\%,$$

where *L*_f_ is the linear change rate, while *L*_1_ and *L*_2_ are the lengths of the samples before and after sintering, respectively.

(5) $$k=\frac{Q\times L}{S\times (T_{1}-T_{2})},$$

where *k* is thermal conductivity, *Q* is the amount of heat flowing per unit time, *L* is the thickness of samples, and *T*_1_ and *T*_2_ are the temperatures at hot and cold face, respectively. S is the area of calorimeter.

## 3. Results and Discussion

### 3.1. Phase Analysis

**Figure 1:**
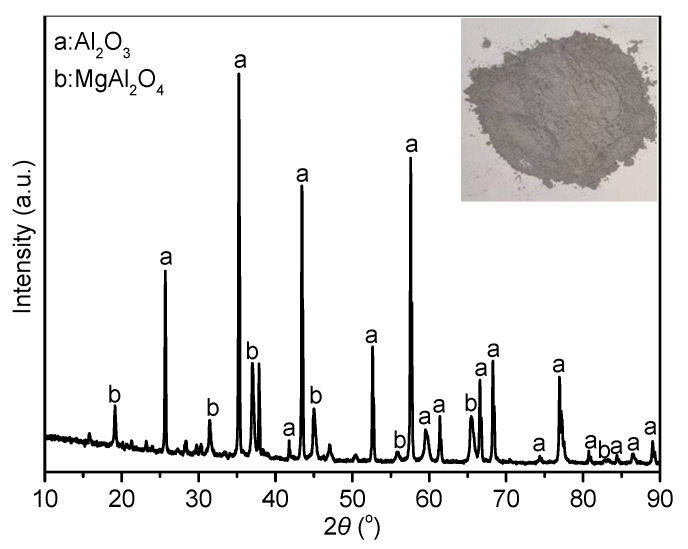
XRD pattern of ADW.

Figure 1 presents the XRD patterns of ADW, wherein the main crystal phases were Al_2_O_3_ (PDF No. 01-071-1124) with the trace of MgAl_2_O_4_ (PDF No. 00-002-1084). However, certain other impurities, such as CaO and SiO_2_ were not detected in the diffractogram, possibly due to the low content, thereby inhibiting their detection. The calculations using Jade 6.0 software show that the unit cell parameter of MgAl_2_O_4_ (a = 8.05959 Å) prominently deviated from the unit cell parameter of stoichiometric MgAl_2_O_4_ spinel (a = 8.0831 Å). When Al_2_O_3_ was solid dissolved in spinel, Al^3+^ (0.057 nm) replaced Mg^2+^ (0.074 nm) with a larger ionic radius, forming Mg^2+^ vacancies, which can easily form pores in the spinel. Moreover, the Al_2_O_3_ was solid-dissolved, lower than the lattice constant. Therefore, the spinel solid solution phase in ADW is an aluminum-rich spinel. Due to the solid solution effect of alumina, the lattice distortion of aluminum-rich spinel was greater than that of the stoichiometric spinel. Meanwhile, the solid solution delays the sintering so that the aluminum-rich spinel has smaller grains and more pores than that of stoichiometric spinel. Chen et al. [22] concluded that the apparent porosity of aluminum-rich spinel samples is significantly higher than that of stoichiometric spinel. Therefore, the ADW containing a certain content of aluminum-rich spinel is light weight and has a porous structure. Due to the lattice defects and smaller grain size and average pore diameter, aluminum-rich spinel has larger specific surface area, increased reactivity, better overall bonding during sintering, and increased strength. Therefore, a certain content of aluminum-rich spinel endows ADW with the characteristic of easy sintering.

**Figure 2:**
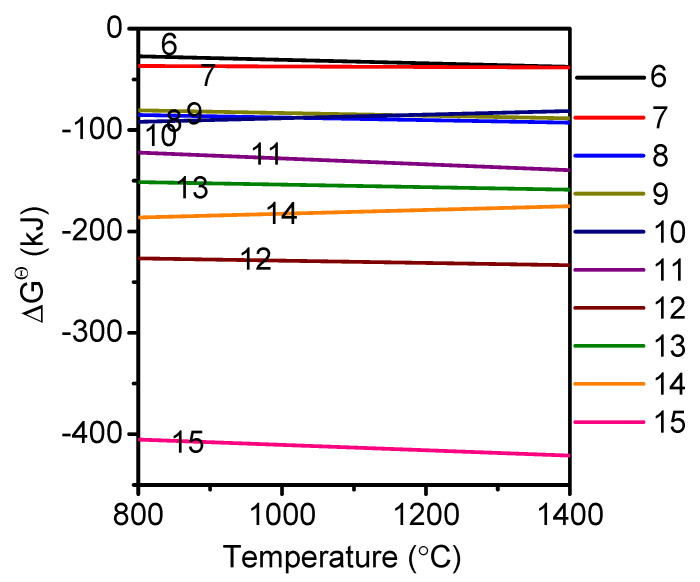
Standard Gibbs energy values corresponding to reactions (6)–(15), as a function of temperature.

**Figure 3:**
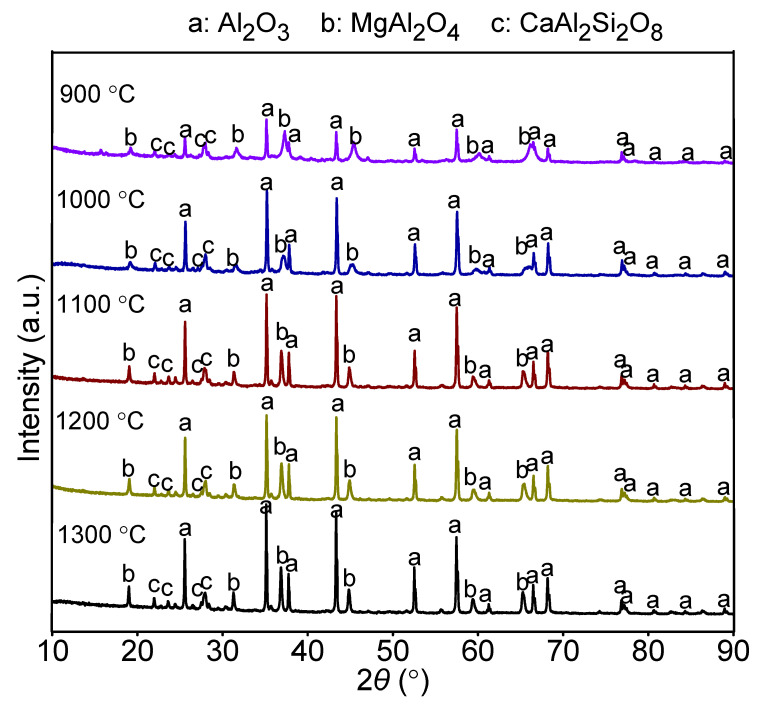
XRD patterns of 85A castable sintered at different temperatures.

The sintering process of castable primarily involves the formation of xCaO-yAl_2_O_3_-zSiO_2_. The possible reactions are shown in Equations (6)–(15), and their standard Gibbs energy values as a function of temperature are presented in Figure 2. As shown, the free energies were negative, signifying that Equations (6)–(15) can occur spontaneously at 900–1300 °C. However, the CaAl_2_Si_2_O_8_ phase was most stable in the CaO-Al_2_O_3_-SiO_2_ system because the formation of the CaAl_2_Si_2_O_8_ phase had the lowest free energy at 900–1300 °C (Equations (11) and (15)). The XRD results of castables sintered in the temperature range of 900–1300 °C are consistent with the thermodynamic calculations. As shown in Figure 3, the main phases were Al_2_O_3_, MgAl_2_O_4_, and CaAl_2_Si_2_O_8_. The peak intensities of these phases increased with the rise in temperature, indicating greater crystallinity at higher temperature.

3 Al_2_O_3_ (s) + 2 SiO_2_ (s) = 3 Al_2_O_3_·2 SiO_2_ (s), (6)

Al_2_O_3_ (s) + MgO (s) = MgAl_2_O_4_ (s), (7)

CaO (s) + Al_2_O_3_ (s) + SiO_2_ (s) = CaAl_2_SiO_6_ (s), (8)

CaO (s) + 1/2 Al_2_O_3_ (s) + 1/2 SiO_2_ (s) = 1/2 Ca_2_Al_2_SiO_7_ (s), (9)

CaO (s) + 1/3 Al_2_O_3_ (s) + SiO_2_ (s) = 1/3 Ca_3_Al_2_Si_3_O_12_ (s), (10)

CaO (s) + Al_2_O_3_ (s) + 2 SiO_2_ (s) = CaAl_2_Si_2_O_8_ (s), (11)

CaO (s) + 1/2 Al_2_O_3_·2 SiO_2_ (s)+ 1/2 Al_2_O_3_ (s) = CaAl_2_SiO_6_ (s), (12)

CaO (s) + 1/4 Al_2_O_3_·2 SiO_2_ (s)+ 1/4 Al_2_O_3_ (s) = 1/2 Ca_2_Al_2_SiO_7_ (s), (13)

CaO (s) + 1/3 Al_2_O_3_·2 SiO_2_ (s) + 1/3 SiO_2_ (s) = 1/3 Ca_3_Al_2_Si_3_O_12_ (s), (14)

CaO (s) + Al_2_O_3_·2 SiO_2_ (s) = CaAl_2_Si_2_O_8_ (s), (15)

**Figure 4:**
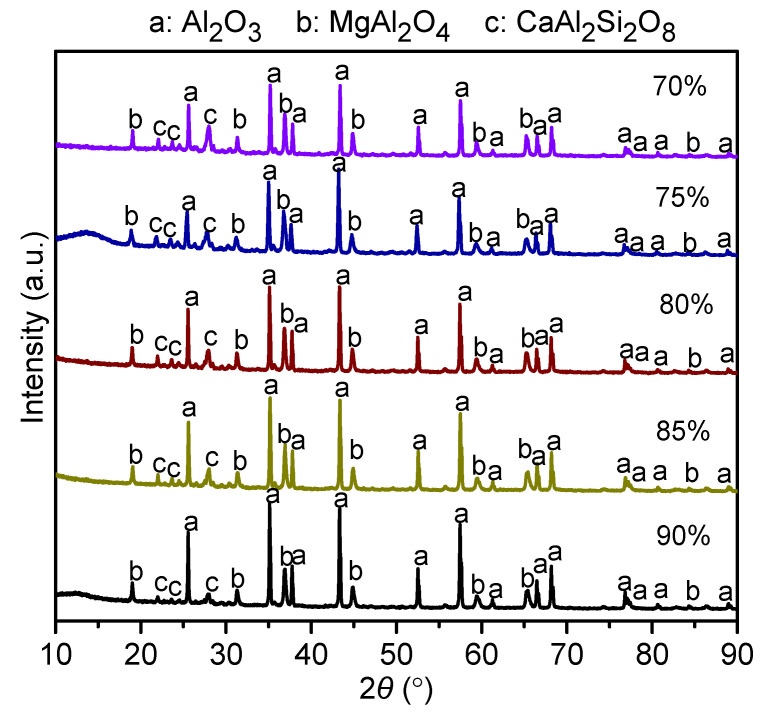
XRD patterns of castables with different amounts of ADW sintered at 1200 °C.

Figure 4 presents the XRD patterns of castables with different amounts of ADW after sintering at 1200 °C. As the ADW amount increased from 70 wt% to 90 wt%, Al_2_O_3_, MgAl_2_O_4_, and CaAl_2_Si_2_O_8_ became the main phases. The Al_2_O_3_ and MgAl_2_O_4_ phases were primarily derived from ADW; therefore, with the rise in ADW, the intensity of Al_2_O_3_ peaks gradually increased. Contrary to the trend of intensity of Al_2_O_3_ peaks, the intensity of CaAl_2_Si_2_O_8_ peaks gradually decreased upon the replacement of clay with ADW.

### 3.2. Microstructure

**Figure 5:**
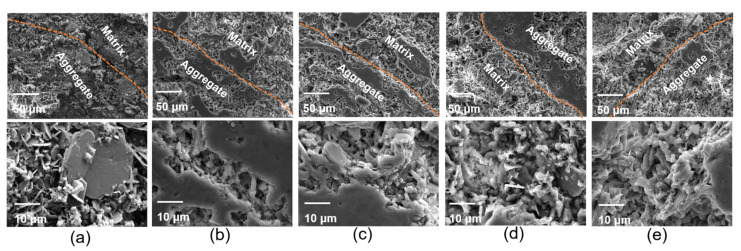
Interface between aggregate and matrix of castables with different amount of ADW calcined at 1200 °C for 2 h: (**a**) 70A, (**b**) 75A, (**c**) 80A, (**d**) 85A, and (**e**) 90A.

Figure 5 shows the microstructure of castable samples after sintering at 1200 °C. Different quantities and dimensions of pores were observed with the enhancement in ADW content. The quantities of pores increased, and then decreased with the rise in ADW, while the dimensions of pores presented the contrary tendency. At 85A sample, it was possible to observe the smallest pores compared to other samples, which were homogeneously distributed and embedded in a crack-free refractory matrix. The fluorine element introduced from ADW could change the morphology of Al_2_O_3_ in the crystallization process and form Al_2_O_3_ flakes. When the content of ADW was increased to 90 wt%, more liquid phase (such as Na_2_F and MgF) was formed; the variation in viscosity affected the pore formation in the sintering process. Hence, the pore dimension was strongly influenced by the rise in salt contents introduced by ADW. Meanwhile, with the increase in ADW content, the CaAl_2_Si_2_O_8_ phase decreased and the aluminum-rich spinel phase increased. The phase evolution influenced the bonding intensity between aggregates and the ceramic matrix. The structures of pores close to the interface between aggregates and the ceramic matrix became smaller, which made them more tightly bound. While, when the ADW content in aggregates was increased to 90 wt%, the low melting point phase introduced by ADW accelerated the sintering process, allowing its densification. However, the compact sample displayed an increase in thermal conductivity.

**Figure 6:**
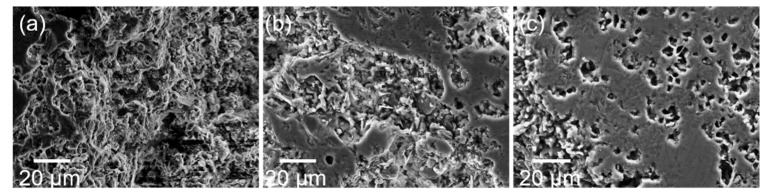
SEM images of 85A sample after sintering at different temperatures: (**a**) 1100 °C, (**b**) 1200 °C, and (**c**) 1300 °C.

The effect of sintering temperature on the microstructure of castables is shown in Figure 6. At 1100 °C, the crystal grains did not precipitate completely due to the relatively lower sintering temperature, and most of them were agglomerated. At 1200 °C, the 85A sample displayed smaller grains and flakes with uniform and small pores; the structure had beneficial impact on the mechanical properties of the castable. When the sintering temperature was further increased to 1300 °C, some low melting point liquid phases (Na_2_F and MgF) emerged, and prominent sintering occurred between the crystal grains, forming uneven and larger clear pores. As per XRD patterns (Figure 3), the intensity of Al_2_O_3_ peaks gradually increased with the rise in sintering temperature, indicating that the Al_2_O_3_ phase displayed greater crystallinity at higher temperatures. Comparing Figure 6b,c, the crystal grains of 85A sample calcined at 1300 °C were larger than those of the samples calcined at 1200 °C.

**Figure 7:**
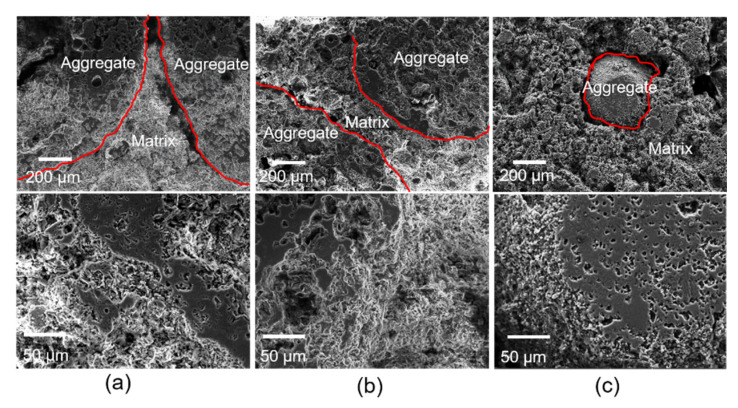
SEM images of the interface between aggregate and matrix: (**a**) 1100 °C, (**b**) 1200 °C, and (**c**) 1300 °C.

SEM images of the interface between aggregate and matrix are shown in Figure 7. The liquid phase increased with the rise in temperature, and the bond between the matrix and aggregates was tighter. However, when the temperature increased to 1300 °C, due to the difference in the brightness of aggregate and the matrix, cracks at the interface were observed. These cracks had a negative impact on the mechanical properties of the castables.

### 3.3. Densification and Mechanical Properties

**Figure 8:**
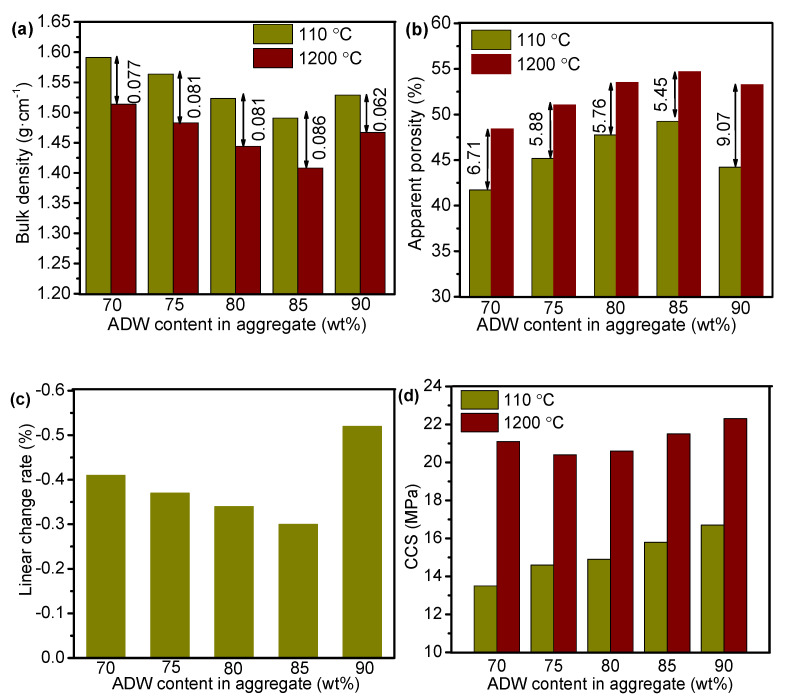
(**a**) Bulk density, (**b**) apparent porosity, (**c**) linear change rate, and (**d**) CCS of the castables with different ADW in aggregates.

Figure 8a,b show the bulk density and apparent porosity, respectively, of the castables before and after sintering at 1200 °C. Compared to green bodies, the castables demonstrated a decrease in bulk density and an increase in apparent porosity as the sintering temperature reached 1200 °C. The abnormal phenomenon was primarily because water and CAC were used during the casting process. Porous structure was formed with the evaporation of physically adsorbed and retained water in the pores and the dehydration of the hydrates present in the samples at high temperatures. Dehydration caused the transformation of hydrated calcium aluminate pastes from the hexagonal metastable form (CAH_10_) to a stable cubic one (C_3_AH_6_) with volume expansion [23]. As shown in Figure 8a, bulk density of the castables before and after sintering decreased first and then increased with the rise in ADW content. The trend of apparent porosity with the increase in ADW content was opposite to that of the bulk density. After sintering at 1200 °C, the lowest value of bulk density (1.41 g/cm^3^) and the highest value of apparent porosity (54.7%) were obtained when the ADW content in aggregate was 85 wt%. With an increase in ADW content, the relative content of clay decreased, which led to a decrease in SiO_2_ and CaO in the castable. These changes in turn caused a decrease in the CaAl_2_Si_2_O_8_ phase content with low melting point after sintering. Accordingly, the content of the Al_2_O_3_-rich MgAl_2_O_4_ phase was enhanced with the rise in ADW content. When the ADW content in the aggregate increased to 90 wt%, the content of impurities (Na_2_F and MgF) from ADW was also increased. These impurities commonly possess low melting point, resulting in volume shrinking and decrease in porosity.

Figure 8c presents the effect of ADW content in aggregates on the linear change rate of castables after sintering at 1200 °C. Values of the linear change rate were negative, indicating that the volume of the samples decreased after sintering at high temperatures. Similar trend was observed with bulk density and smallest linear change rate was achieved when the ADW content in aggregate was 85 wt%. These results are consistent with the change in microstructure shown in Figure 6. The Al_2_O_3_-rich MgAl_2_O_4_ phase increased with the rise in ADW content, which can reduce the shrinkage effect caused by sintering. Meanwhile, the CaAl_2_Si_2_O_8_ phase decreased because CaO and SiO_2_ decreased. With further enhancement in ADW content, low melting point phase induced by ADW increased, leading to a rise in the liquid phase at high temperature. The liquid phase filled the pores generated with the formation of aluminum-rich spinel, causing an increase in volume shrinkage. Microstructural changes and phase evolutions often affect the physical and mechanical behavior of the castables during the sintering process. Figure 8d shows CCS of the castables with different ADW content in aggregates before and after sintering. Compared to green samples, the CCS values were increased after sintering at 1200 °C. Notably, the apparent porosity and CCS of castables were increased simultaneously after sintering. The possible reason for the phenomenon could be that, even though water evaporation led to the formation of a porous structure, the ceramic phases connected with each other under the action of high temperature. The CCS of castables was influenced by many factors, among which porosity and bonding degree between aggregate and matrix had a more significant impact. The CCSs of 75A and 80A samples were lower than that of the 70A sample due to the higher porosities. However, the CCS was increased at the 85A sample even though it possessed the highest porosity (Figure 8b), which could be attributed to the strong aggregate-matrix bonding (Figure 5).

**Figure 9:**
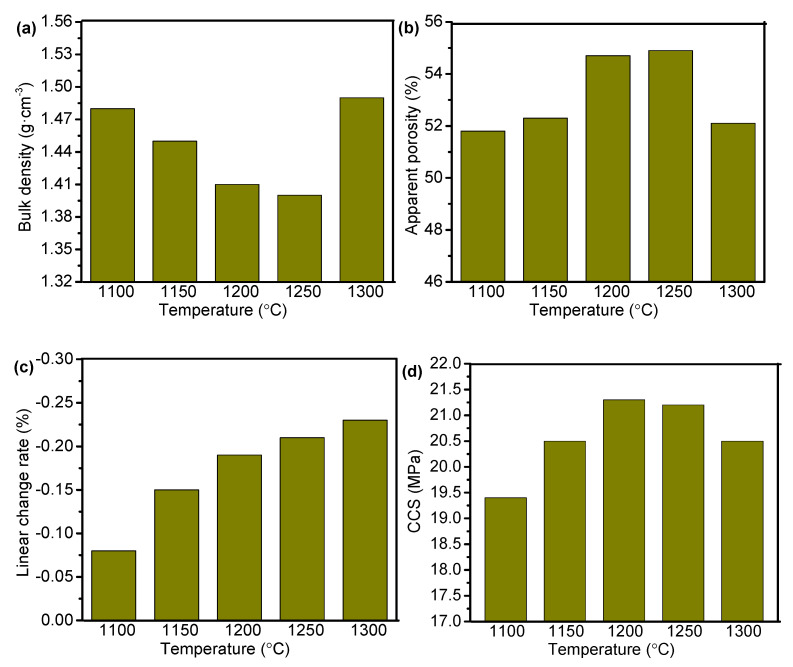
(**a**) Bulk density, (**b**) apparent porosity, (**c**) linear change rate, and (**d**) CCS of the 85A castables after sintering at different temperatures.

**Figure 10:**
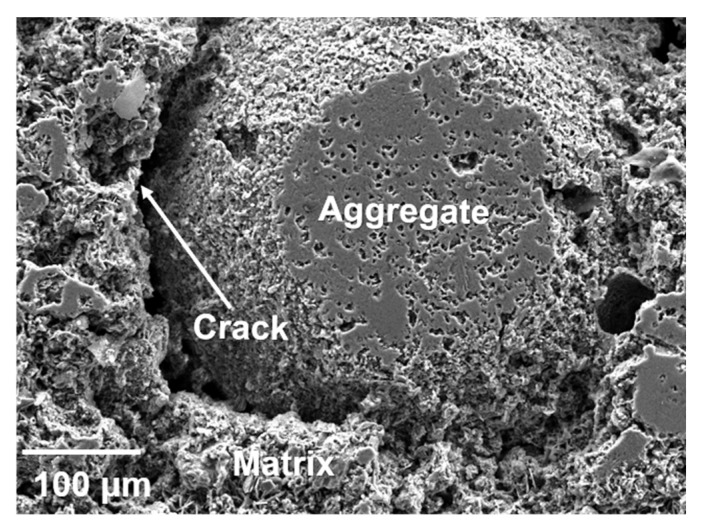
Aggregate-matrix interface after sintering at 1300 °C.

Figure 9a–d present the bulk density, apparent porosity, linear change rate, and CCS of 85A castables as a function of temperature. The bulk density of the castable decreased first and then increased with the rise in sintering temperature from 1100 °C to 1300 °C. Contrary to the trend of BD, with the rise in sintering temperature, the apparent porosity and CCS of the castable increased first and then decreased. When the temperature sequentially increased to 1300 °C, the liquid phase increased and filled a fraction of pores. Meanwhile, higher the temperature, faster was the mass transfer rate, which resulted in rapid sintering of the castable. These two effects increased bulk density and reduced porosity. As per Figure 9c, the linear change rates were increased with the rise in temperature. Moreover, with the increase in temperature, the CCS of samples increased first, and then decreased. A similar variation trend was observed between apparent porosity and CCS (Figure 9b,d). As mentioned earlier, the apparent porosity and the bonding degree between the aggregate and the matrix significantly affected the CCS. The 85A sample has a higher apparent porosity and CCS values at 1200 °C and 1250 °C than the sample at 1100 °C and 1150 °C because the bonding degree increased between the aggregate and the matrix. The unexpected decrease in CCS at 1300 °C can primarily be attributed to the cracks between the aggregates and the ceramic matrix (Figure 10).

### 3.4. Thermal Conductivity

**Figure 11:**
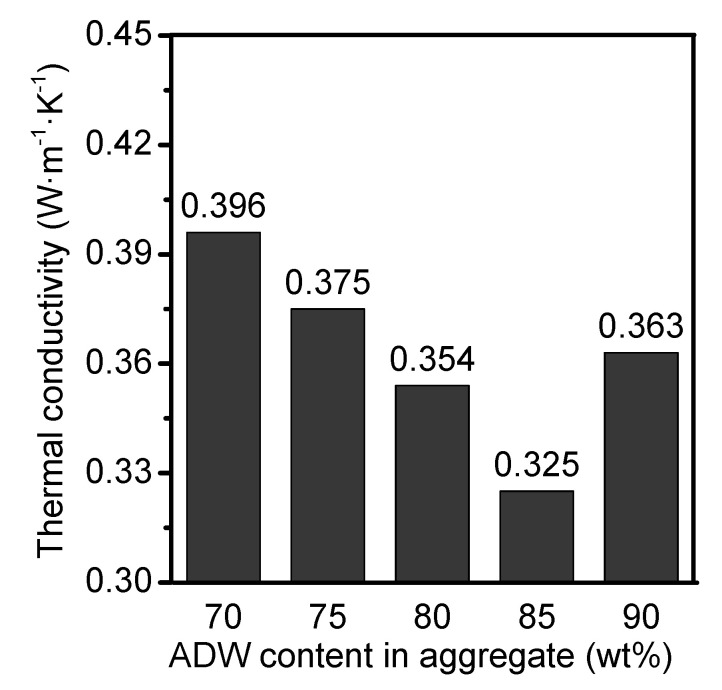
Thermal conductivities of castables with different ADW contents after sintering at 1200 °C.

**Figure 12:**
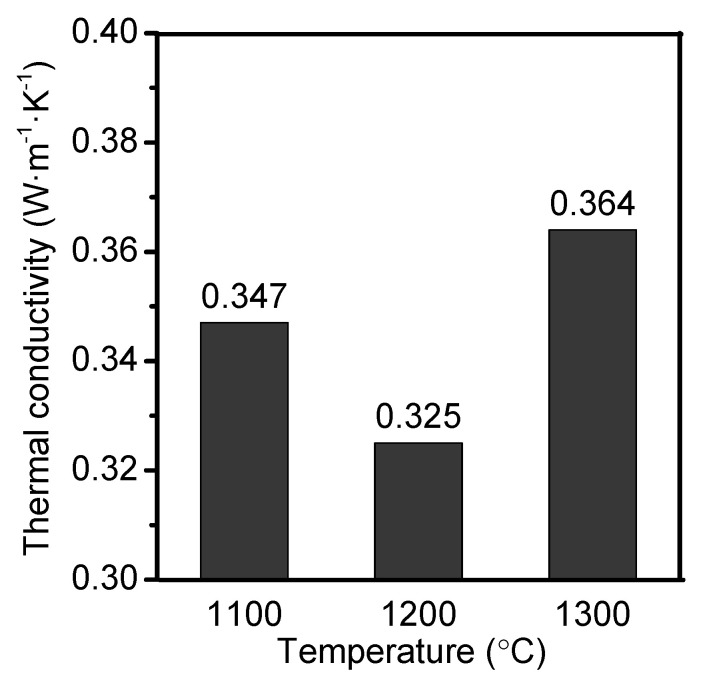
Thermal conductivities of A85 castables after sintering at different temperatures.

Figure 11 shows the thermal conductivities of castables with different ADW contents after sintering at 1200 °C. With the rise in ADW content, the thermal conductivity decreased first and then increased. Figure 12 shows the relationship between thermal conductivity and ADW contents in aggregates. The as-prepared ADW-containing porous refractory castables displayed relatively low thermal conductivity in the range of 0.325–0.396 W·m^−1^·K^−1^. In general, thermal conductivity of a material with a solid state is larger than that of air gas (0.024 W·m^−1^·K^−1^ at 0 degrees and 0.031 W·m^−1^·K^−1^ at 100 degrees). Therefore, the heat conduction of porous ceramics is mainly determined by the degree of porosity and the pore size distribution. Based on the above discussed Figure 8b and Figure 9b, the A85 sample was found to have highest apparent porosity after sintering at 1200 °C, resulting in extremely low thermal conductivity (0.325 W·m^−1^·K^−1^) as compared to other samples (Figure 12).

Table 3 compares the performance of 85A castables and national standards (GB/T22590-2008) of refractory castables for reheating furnaces. As shown, the properties of the 85A sample, such as bulk density, cold crushing strength, thermal conductivity, linear change rate, and operating temperature fulfilled the requirements. These results suggest that partial replacement of clay by ADW is feasible for the fabrication of Al_2_O_3_-SiO_2_-rich castables used as reheating furnaces.

## 4. Conclusions

Al_2_O_3_-SiO_2_-rich castable refractories were successfully prepared by using ADW partial substitution of clay. The effects of the ADW content and sintering temperature on the microstructure and phase evolution of the castables were explored. Moreover, the relationship among the phase and microstructure and the physical and mechanical properties, and thermal conductivity of the castables was developed. The results show that, when the ADW content in aggregate was 85 wt%, the Al_2_O_3_-SiO_2_-rich castable has desired properties. For instance, the bulk density, cold crushing strength at 110 °C and 1000 °C, thermal conductivity, and linear change rate were 1.41 g·cm^−3^, 15.8 MPa, 21.5 MPa, 0.325 W·m^−1^·K^−1^, and −0.31%, respectively. These properties fulfil the requirements of national standards of refractory castables for reheating furnaces. The use of ADW to prepare Al_2_O_3_-SiO_2_-rich castables is a new attempt, which can not only reduce the waste discharge and improve the environment, but also reduce the use of natural raw materials and enhance economic benefits.

## Figures and Tables

**Table 1:** XRF results of starting materials (wt%).

	Al_2_O_3_	MgO	SiO_2_	CaO	K_2_O	Na_2_O	Fe_2_O_3_	TiO_2_	MnO	F
Aluminum dross	82.50	8.65	1.75	2.32	0.43	1.48	0.38	0.92	-	1.57
Clay	13.21	2.93	75.73	5.64	0.45	0.03	0.97	0.88	0.16	-

**Table 2:** Chemical composition of castable (wt%).

		70A	75A	80A	85A	90A
Castable aggregate	ADW	35.0	37.5	40.0	42.5	45.0
	Clay	15.0	12.5	10.0	7.5	5.0
Al_2_O_3_ powder		10	10	10	10	10
Silica fume		5	5	5	5	5
Clay powder		5	5	5	5	5
ADW powder		20	20	20	20	20
Calcium aluminate cement		10	10	10	10	10

**Table 3:** Properties comparison of as-prepared Al_2_O_3_-SiO_2_-rich castable and the national standard (GB/T22590-2008).

		85A	ZJQ120-1.5
Bulk density (g/cm^3^)	110 °C × 24 h	1.41	≤1.5
Cold crushing strength (MPa)	110 °C × 24 h	15.80	≥6
Cold crushing strength (MPa)	1200 °C × 3 h	21.50	≥15
Thermal conductivity (W·m^−1^·K^−1^)	-	0.325	≤0.75
Linear change rate (%)	1200 °C × 3 h	−0.31	−0.5~0

## Data Availability

Data available in a publicly accessible repository.
